# Folium *Ginkgo* extract and tetramethylpyrazine sodium chloride injection (Xingxiong injection) protects against focal cerebral ischaemia/reperfusion injury via activating the Akt/Nrf2 pathway and inhibiting NLRP3 inflammasome activation

**DOI:** 10.1080/13880209.2021.2014895

**Published:** 2022-01-21

**Authors:** Ting Zhu, Bin-Yu Fang, Xiang-Bao Meng, Shu-Xia Zhang, Hong Wang, Ge Gao, Fei Liu, Yu Wu, Jin Hu, Gui-Bo Sun, Xiao-Bo Sun

**Affiliations:** a Beijing Key Laboratory of Innovative Drug Discovery of Traditional Chinese Medicine (Natural Medicine) and Translational Medicine, Beijing, China; b Key Laboratory of Bioactive Substances and Resources Utilization of Chinese Herbal Medicine, Ministry of Education, Beijing, China; c Key Laboratory of New Drug Discovery Based on Classic Chinese Medicine Prescription, Beijing, China; d NMPA Key Laboratory for Research and Evaluation of Pharmacovigilance, Institute of Medicinal Plant Development, Chinese Academy of Medical Sciences & Peking Union Medical College, Beijing, China; e Institute of Neuroregeneration and Neurorehabilitation, Qingdao University, Qingdao, China; f Harbin University of Commerce, Harbin, China; g Sihuan Pharmaceutical Holdings Group Ltd, Beijing, China

**Keywords:** Ischaemic stroke, apoptosis, inflammation, oxidative stress

## Abstract

**Context:**

Folium *Ginkgo* extract and tetramethylpyrazine sodium chloride injection (Xingxiong injection) is a compound preparation commonly used for treating cerebral ischaemia/reperfusion injury in ischaemic stroke in China. However, its potential mechanisms on ischaemic stroke remain unknown.

**Objective:**

This study explores the potential mechanisms of Xingxiong injection *in vivo* or *in vitro.*

**Materials and methods:**

Sprague-Dawley (SD) rats were randomly assigned to five groups: the sham (normal saline), the model (normal saline) and the Xingxiong injection groups (12.5, 25 or 50 mL/kg). The rats were subjected to 2 h of middle cerebral artery occlusion (MCAO) followed by reperfusion for 14 d. Xingxiong injection was administered via intraperitoneal (i.p.) injection immediately after ischaemia induction for 14 d. Afterwards, rats were sacrificed at 14 d induced by administration of Xingxiong injection.

**Results:**

Xingxiong injection significantly reduces infarct volume (23%) and neurological deficit scores (93%) compared with the MCAO/R group. Additionally, Xingxiong injection inhibits the loss in mitochondrial membrane potential (43%) and reduces caspase-3 level (44%), decreases NOX (41%), protein carbonyl (29%), 4-HNE (40%) and 8-OhdG (41%) levels, inhibits the expression of inflammatory factors, such as TNF-α (26%), IL-1β (34%), IL-6 (39%), MCP-1 (36%), CD11a (41%) and ICAM-1 (43%). Moreover, Xingxiong injection can increase p-Akt/Akt (35%) and Nrf2 (47%) protein expression and inhibit NLRP3 (42%) protein expression.

**Conclusions:**

Xingxiong injection prevents cerebral ischaemia/reperfusion injury via activating the Akt/Nrf2 pathway and inhibiting NLRP3 inflammasome. These findings provide experimental evidence for clinical use of drugs in the treatment of ischaemic stroke.

## Introduction

Stroke is a leading cause of mortality and serious long-term disability worldwide, which afflicted over 80 million people globally by 2016 (Zhao et al. 2018). Of all stroke events, ischaemic stroke (caused by decreased blood flow to the brain) accounts for approximately 87% in nature. The standard of care for clinical management of acute ischaemic stroke is intervention with intravenous thrombolysis via the recombinant tissue plasminogen activator (r-tPA). However, r-tPA therapy is less than ideal due to its the narrow treatment window, contraindications and complications, only 1–5% of patients currently receive tPA treatment (Aiyagari et al. 2004). Therefore, alternative therapeutic options are required.

Laboratory research has shown ischaemic stroke in response to infarct volume reduction and neurological dysfunction in a MCAO/R rat model. During cerebral ischaemia, deficiency of glucose and oxygen causes ATP depletion, which results in membrane depolarization and further induces multiple mechanisms including neuronal excitotoxicity, acidotoxicity, inflammation response, oxidant injury and mitochondrial response. Membrane depolarization also promotes intracellular calcium release, initiating the activation of apoptosis, necrosis and autophagy signalling pathways (Zhou et al. 2018). In addition, continuous calcium release accelerates reactive oxygen species (ROS) production, which triggers inflammatory responses, consequently increasing blood–brain barrier (BBB) permeability and resulting in neuronal death (Szydlowska and Tymianski 2010). Taken together, the pathophysiology of ischaemic stroke involves complicated molecular mechanisms and pathways. Drugs with only one single component generally cannot target all the above pathways; therefore, they have less efficacy and may have some adverse effects. Due to the multi-component and multi-targeted features as well as their higher safety, the experimental research and clinical application of multi-targeted medicine has exhibited great advantage in the treatment of ischaemic stroke in recent years (Zhong et al. 2005; Wang et al. 2017; Zhu et al. 2021b).

Folium *Ginkgo* extract and tetramethylpyrazine sodium chloride injection (Xingxiong injection) was approved in 2004 in China, where it is widely used for treating ischaemic cardiovascular or cerebrovascular diseases, peripheral circulatory disorder, hypertension, hyperlipidaemia, etc. The therapeutic effects of this Xingxiong injection were validated (Yang and Sun 2007), especially for cerebral infarction. The active ingredients in folium *Ginkgo* extract, flavonoids and ginkgolides, were reported to improve blood circulation and memory (Cheuvront and Carter 2003; Amieva et al. 2013). Ligustrazine phosphate was reported to exhibit antioxidant, anti-apoptotic and calcium antagonistic effects (Zhu et al. 2005; Li et al. 2010). However, the therapeutic mechanism of Xingxiong injection as a compound preparation is still not well understood due to a lack of scientific evidence indicating whether it has beneficial roles in the treatment of cerebral ischaemia/reperfusion injury.

The present study investigated the protective effects of the Xingxiong injection in a MCAO/R rat and OGD/R neuron model to clarify its potential mechanism from the aspects of anti-apoptosis, antioxidation and anti-inflammatory.

## Materials and methods

### Materials

Xingxiong injection was obtained from Honghe Pharmaceutical Co., Ltd. (Jilin, China). Triphenyltetrazolium chloride (TTC), poly-L-lysine, dimethyl sulphoxide (DMSO) were purchased from Sigma-Aldrich (St. Louis, MO). Dulbecco’s modified Eagle’s medium/F12, foetal bovine serum (FBS), neurobasal medium and B27 were purchased from Gibco (Brooklyn, NY). 5,5′,6,6′-Tetrachloro-1,1′,3,3′-tetraethyl benzimidazolyl carbocyanine iodide (JC-1) was purchased from Beyotime Biotechnology (Shanghai, China). Caspase-3 fluorescence staining kit was purchased from BioVision (Milpitas, CA). All ELISA kits were purchased from Expandbio (Beijing, China).

### Animals

Male Sprague-Dawley (SD) rats (250–280 g) were purchased from Beijing Vital River Laboratories (Beijing, China) and used in this study. All animal care and experimental protocols were approved by the Institutional Animal Care and Use Committee of the Chinese Academy of Medical Sciences & Peking Union Medical College (no. SYXK 2017-0020). All efforts were made to minimize the number of animals used and to ensure minimal suffering. All animals were maintained in ventilated cages at 20–25 °C and relative humidity (30–50%) with a 12 h light/dark cycle, and given free access to food and water. All animals used in this study showed clinically normal, free of apparent infection or inflammation or neurological deficits before the experimental procedures.

### MCAO surgery

SD rats were anaesthetized with ketamine (80 mg/kg) and xylazine (10 mg/kg) intraperitoneally (i.p.) and underwent middle cerebral artery occlusion (MCAO) procedure. Cerebral ischaemia–reperfusion (I/R) was induced in rats through middle cerebral artery occlusion/reperfusion (MCAO/R) by following a previously described method (Zhu et al. 2020, 2021c). The occlusion lasted for 2 h until reperfusion. The rats were manipulated using the same technique in the sham group, but the MCA was not occluded. The rectal temperature of rats was maintained at 37 ± 0.5 °C during the entire procedure by using a heating blanket (Sunbeam, Boca Raton, FL).

### Drug treatment

The rats were randomly assigned to five experimental groups: (1) a sham group; (2) an MCAO/R group injected with an isovolumetric solvent (saline) for 14 days; groups injected with (3) 12.5, (4) 25 and (5) 50 mL/kg/day Xingxiong injection (Honghe Pharmaceutical Co., Ltd., Jilin, China) for 14 days after MCAO. For drug administration, Xingxiong injection or vehicle (0.9% normal saline) was given immediately after MCAO surgery. At 14 d post-injection, some brain tissues and serum were collected in −80 °C for subsequent experiments.

### TTC staining

Triphenyltetrazolium chloride staining was conducted 7 d post-stroke based on the methods described previously (Zhu et al. 2020, 2021a). Cerebral infarct size was measured using Image-Pro Plus version 5.0 analysis software. Infarct areas of all sections were added to derive the total infarct area, which was multiplied by the thickness of the brain sections to obtain the infarct volume. The corrected infarct volume was calculated as before to compensate for the effect of brain oedema. Repeat three times for each group.

### Histopathology staining

Histopathological staining (14 d postreperfusion) was conducted based on previously described methods (Wang et al. 2016). The brain samples were embedded in paraffin and coronally dissected into 5 μm thick sections. Then, paraffin sections were stained with H&E and Nissl staining to reveal the histopathological lesions.

### TUNEL staining

Neuronal apoptosis was detected by TUNEL staining with the neural marker NeuN (Abcam, Cambridge, UK). The brain slices (*n* = 3 for each group) were incubated with proteinase K (20 μg/mL) for 15 min and then stained using a TUNEL kit (Beyotime Institute of Biotechnology, Shanghai, China) according to the manufacturer’s instructions. The nucleus was separately stained using DAPI (0.1 μg/mL). Images of the TUNEL-positive neuronal cells were captured using a fluorescence microscope (Carl Zeiss, Oberkochen, Germany).

### Enzyme-linked immunosorbent assay

Right cortex tissues, serum and cell lysis solution were collected from each sample, and the total protein was extracted using a protein extraction reagent supplemented with protease and phosphatase inhibitor cocktails (ComWin Biotech, Beijing, China). The total protein concentration of tissue and cell samples was determined by a BCA kit (ComWin Biotech, Beijing, China). The expression level of TNF-α, IL-1β, IL-6, MCP-1, CD11a, ICAM-1, caspase-1, NOX, PC, 4-HNE and 8-OHdG was assessed using enzyme-linked immunosorbent assay (ELISA) kits according to the previous method (Xie et al. 2019). All ELISA kits were purchased from Expandbio (Beijing, China).

### Western blot analysis

Western blot analysis was conducted as previously described (Xie et al. 2019; Zhu et al. 2021d). Right cortex tissues were collected from each rat (*n* = 3), and the total protein was extracted using a protein extraction reagent supplemented with protease and phosphatase inhibitor cocktails (ComWin Biotech, Beijing, China). The total protein concentration of each sample was determined by a BCA kit (ComWin Biotech, Beijing, China). Equal amounts of protein were separated using SDS-polyacrylamide gel electrophoresis and transferred to nitrocellulose membranes. The membranes were blocked before being incubated overnight at 4 °C with the appropriate primary antibodies against the following proteins: NLRP3 (A5652, 1:1000), IL-1β (Ab16288, 1:1000), Nrf2 (A0674, 1:1000), HO-1 (A1346, 1:1000), Akt (CST4685, 1:1000), p-Akt (CST4060T, 1:1000) and β-actin (EXP0036 F, 1:2000). Then, the membranes were washed three times and incubated with the appropriate secondary antibodies. The blots were visualized using enhanced chemiluminescence western blot detection kits (ComWin Biotech, Beijing, China). The blot densities were measured by Image J software (Bethesda, MD).

### Cell culture

Primary cortical neurons were separated from cerebral cortices of rat foetuses (embryonic day 18) and were cultured on poly-l-lysine-coated plates referencing to a previously method with some modifications (Meng et al. 2014a). Dissociated cortical neurons grown in neurobasal medium supplemented with 2% B27 and 2 mM l-glutamine and changed every other day by replacing half of the medium. Cortical neurons were seeded with a density of 1 × 10^5^ cells/cm^2^ for neurite outgrowth and differentiation. The neuron purity identification was assessed by immunofluorescence using the antibody against microtubule-associated protein-2 (MAP2), a marker of terminal neuronal differentiation. Dulbecco’s modified Eagle’s medium/F12, FBS, neurobasal medium and B27 were purchased from Gibco (Brooklyn, NY).

### Oxygen-glucose deprivation/re-oxygenation

OGD/R was conducted with primary cortical neurons grown to mimic cerebral I/R injury *in vitro*. This technique was conducted according to previously described method with minor modification (Lu et al. 2011; Meng et al. 2014b). Briefly, primary cortical neurons cultured in glucose-free Locke’s medium under hypoxia for 2 h and then removed from the anaerobic chamber (TYPE c; Coy Laboratory Products, Inc., Grass Lake, MI) to a normoxic environment with the medium replaced by normal medium and were allowed to re-oxygenate for 24 h.

### Detection of caspase-3 activity

The activity of caspase-3 was detected according to the previous method (Lu et al. 2019).

### Mitochondrial membrane potential determination

5,5′,6,6′-Tetrachloro-1,1′,3,3′-tetraethyl benzimidazolyl carbocyanine iodide (JC-1) staining was used to determine the changes in mitochondrial membrane potential by Multiscan Spectrum analysis. Cells were pre-treated with concentrations of Xingxiong injection (1, 2 and 4 μL/mL). The cells were challenged with OGD/R after rinsing twice with PBS. After above processes, the cells were loaded 2 mM JC-1 (Beyotime Biotechnology, Shanghai, China) and incubated for 30 min at 37 °C in the dark. Fluorescence intensity was detected at 514/585 nm excitation wave length and 529/590 nm emission wavelength for monomer/J-aggregates (green light/red light) using Multiscan Spectrum (Tecan, Männedorf, Switzerland) (Meng et al. 2014b).

### Statistical analysis

Data were obtained from at least three independent experiments and expressed as means ± standard deviation (SD). Data were analysed using one-way ANOVA, followed by Tukey’s test with GraphPad Prism 6.0 (SPAA, Inc., Chicago, IL), and the graphical presentation of data was also undertaken with GraphPad Prism (SPAA, Inc., Chicago, IL). *p* < 0.05 was considered statistically significant.

## Results

### Reduction of cerebral infarction volumes and neurologic deficits by Xingxiong injection in rats

**Figure 1.**
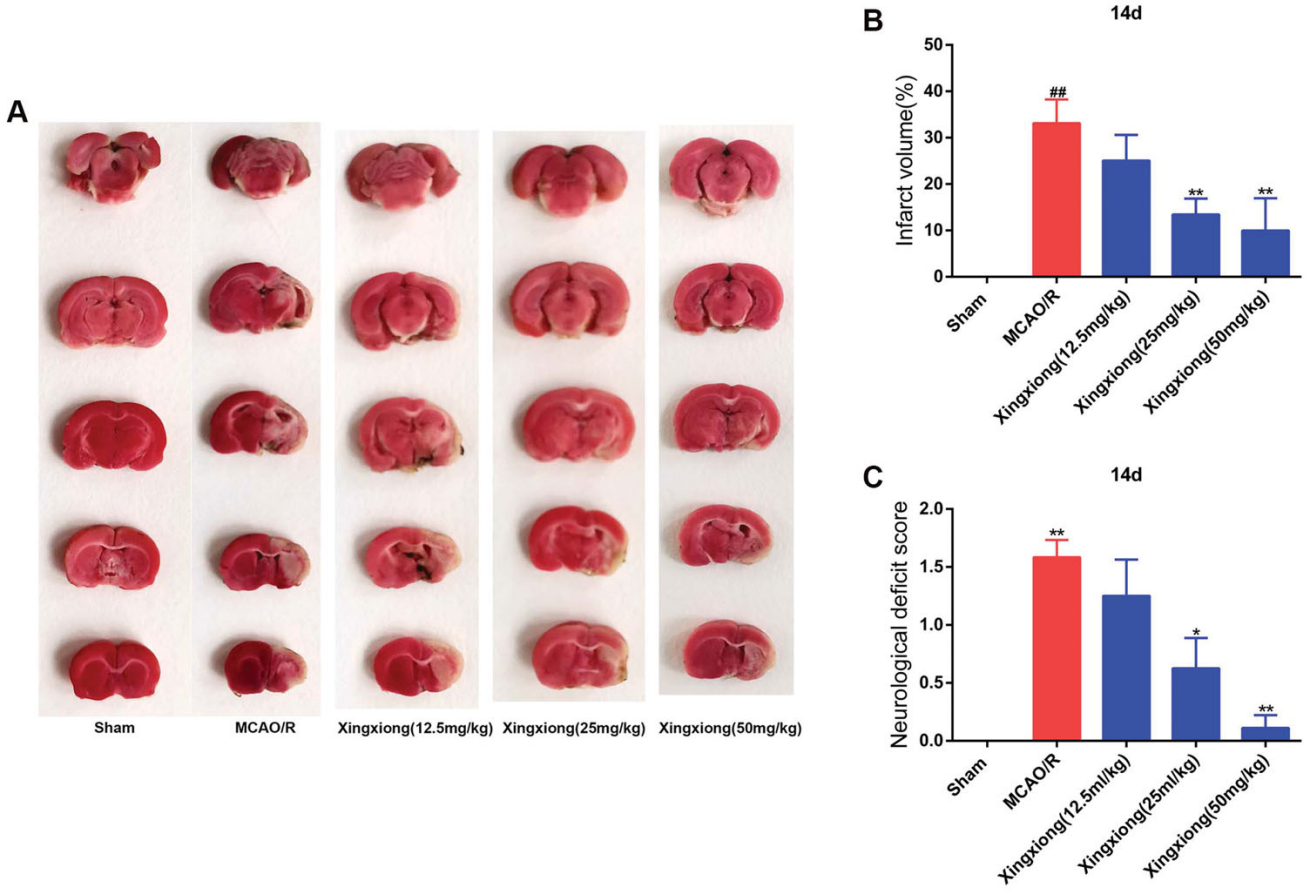
Xingxiong injection reduces the infarction volume and neurologic deficits in rats subjected to MCAO. (A) Effects of Xingxiong injection on infarction volumes (*n* = 3). (B) Quantitative analysis of cerebral infarct volumes. (C) Neurological score in rats with MCAO/R at 14 d after reperfusion (*n* = 3). Data are expressed as the mean ± SD and were analysed by ANOVA. ^##^*p* < 0.01 vs. sham group; *p < 0.05 and **p < 0.01 vs. MCAO/R group.

The cerebral infarction was examined 14 d after MCAO surgery performed on the right side in rats through TTC staining. Figure 1(A,B) shows typical photographs and quantitative data of TTC staining of the sham group, MCAO/R group, Xingxiong injection (12.5, 25 and 50 mL/kg). Administration of Xingxiong injection produced reductions in the infarct volumes compared to MCAO/R group. After treatment of Xingxiong injection at the dosages of 25 and 50 mL/kg for 14 d, infarction volumes significantly decrease than those in the MCAO/R group. In addition, Figure 1(C) shows a visible improvement in neurologic deficit in Xingxiong group.

### Suppression of ischaemia-induced neuronal injury by Xingxiong injection in rats

**Figure 2.**
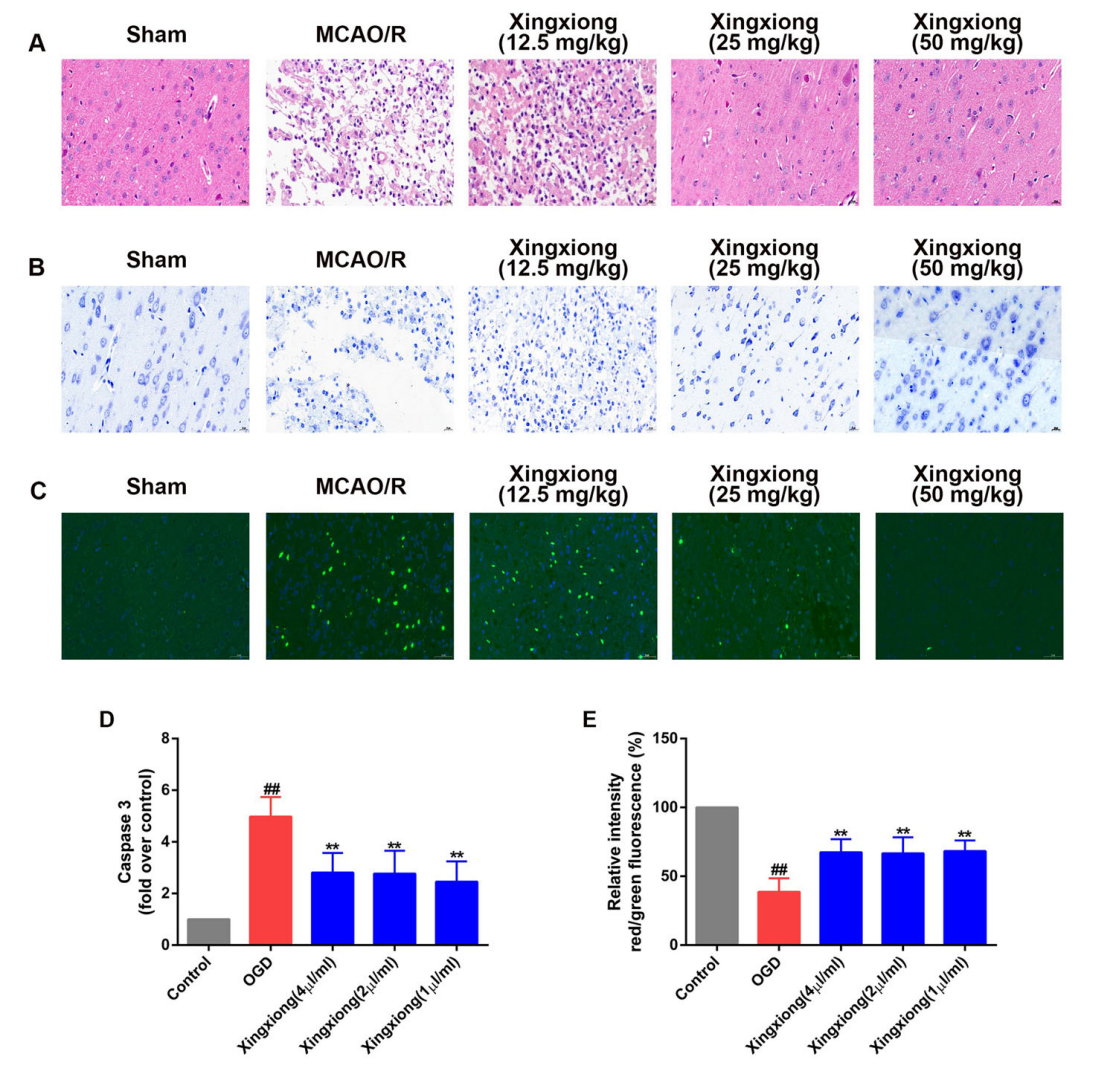
Xingxiong injection reduces neuronal injury and apoptosis of rats with MCAO/R and primary cortical neurons with OGD/R. (A) H&E staining in the cortex region in different groups. Scale bar: 200 μm, *n* = 3; (B) Nissl staining in the cortex region in different groups. Scale bar: 200 μm, *n* = 3; (C) immunofluorescence for TUNEL-positive neurons in the cortex, *n* = 3. Scale bar: 50 μm. (D) Caspase-3 activity of primary cortical neurons with OGD/R was measured using a fluorometric assay. (F) Primary cortical neurons were dyed with JC-1 and then detected using a fluorescence microscope. Data are expressed as the mean ± SD and were analysed by ANOVA. ^##^*p* < 0.01 vs. control group; **p < 0.01 vs. OGD/R group.

H&E-stained slides of rat brain sections were detected under a light microscope (Figure 2(A)). Rats in sham group have many neurons present in the pyknotic nuclei, whereas MCAO/R rats have pale nuclei in the cortex regions. After treatment of Xingxiong injection at the dosages of 25 and 50 mL/kg for 14 d, pyknotic nuclei significantly reduce than those in the MCAO/R group. In addition, Nissl staining shows that most neurons in the cortex region in the MCAO/R group had a shrunken phenotype, weak staining and were scattered irregularly. Similarly, Xingxiong injection at the dosages of 25 and 50 mL/kg for 14 d results in strong staining and regularly arranged neurons in the cortex.

### Suppression of ischaemia/hypoxia-induced apoptosis by Xingxiong injection *in vivo* and *in vitro*

To further explore the neuropathological alterations in ischaemic brains from all groups, neuronal apoptosis in the ischaemic brain tissues was examined after 14 d reperfusion following 2 h of MCAO. The immunofluorescent results showed that the MCAO/R-induced rats exhibited more TUNEL-positive neurons in the ischaemic cortex regions than the sham group (Figure 2(C)). However, Xingxiong injection at the dosages of 25 and 50 mL/kg treatment decreased the number of TUNEL-positive neurons in the ischaemic brain.

The expression of active caspase-3 of the primary cortical neurons separated from E18 SD was detected. The result showed that the expression of active caspase-3 was up-regulated in the OGD-treated group compared with the control group. However, the expression levels of caspase-3 were significantly suppressed by the treatment of Xingxiong injection (Figure 2(D)). In addition, mitochondrial membrane potential of the primary cortical neurons, detected with the ratio of red and green, was suppressed by the treatment of OGD while the effects were dramatically reversed by treatment of Xingxiong injection (Figure 2(E)).

### Suppression of ischaemia/hypoxia-induced oxidative stress by Xingxiong injection *in vivo* and *in vitro*

**Figure 3.**
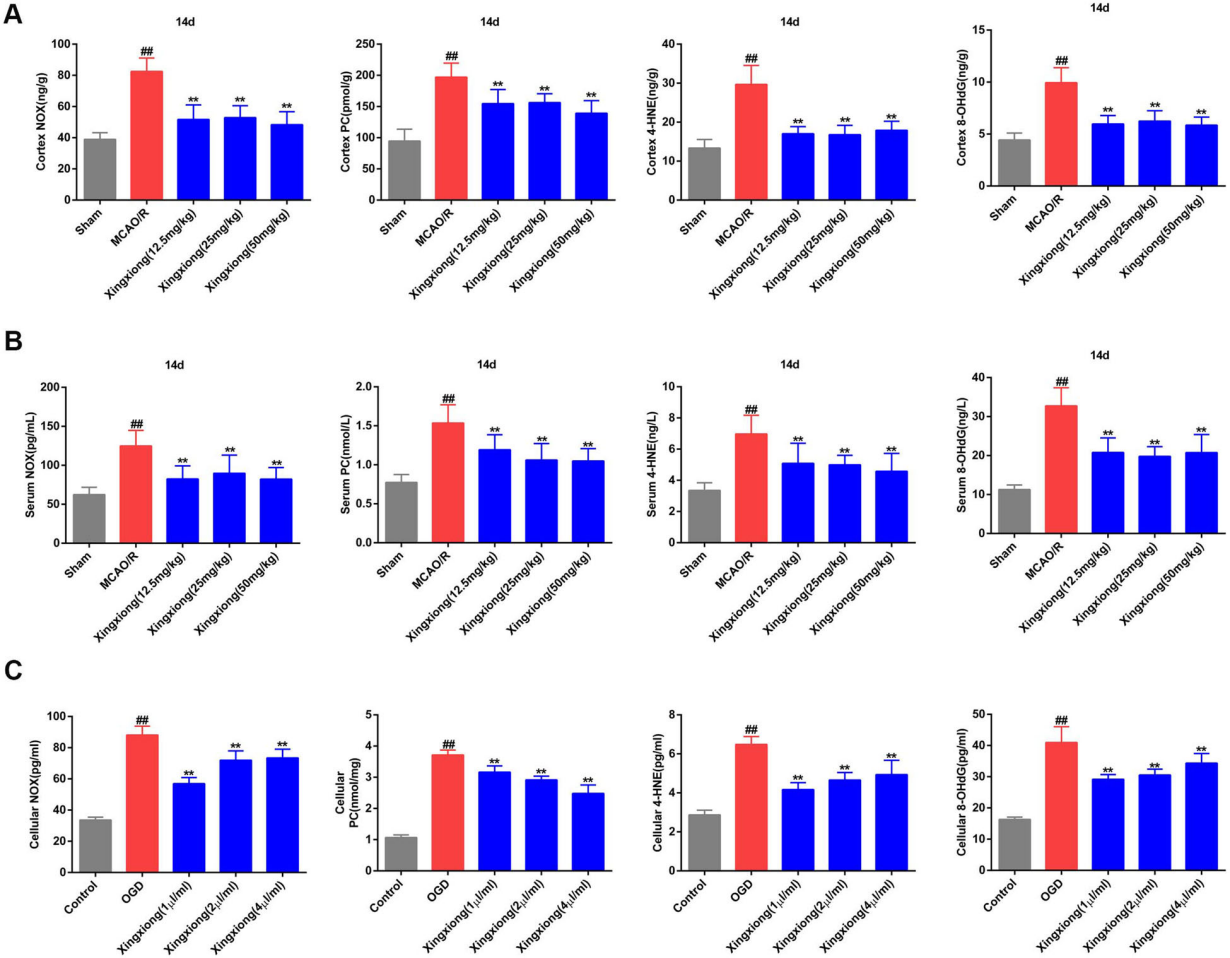
Xingxiong injection inhibits oxidative stress in rat cortex and serum with MCAO/R and in primary cortical neurons with OGD/R. Effect of Xingxiong injection treatment on NOX, PC, 4-HNE and 8-OHdG in rat cortex (A) and serum (B) with MCAO/R (*n* = 8) and in primary cortical neurons (C) with OGD/R (*n* = 3). Data are expressed as the mean ± SD and were analysed by ANOVA. ^##^*p* < 0.01 vs. sham or control group; **p < 0.01 vs. MCAO/R and OGD/R group.

Results of ELISA of ischaemia/hypoxia-induced oxidative stress *in vivo and in vitro* are shown in Figure 3. Compared with the sham group, MCAO/R treatment resulted in a significantly increase in the level of NOX, PC, 4-HNE and 8-OHdG in rat cortex (Figure 3(A)) and serum (Figure 3(B)) after MCAO surgery for 14 d. Furthermore, these factors also displayed a notable increase in OGD-treated primary cortical neurons (Figure 3(C)). However, Xingxiong injection substantially downregulated the levels of NOX, PC, 4-HNE and 8-OHdG in the serum and cortex of MCAO-treated rats and in OGD-treated primary cortical neurons.

### Xingxiong injection reduces oxidative stress by activating the Akt/Nrf2/HO-1 pathway

**Figure 4.**
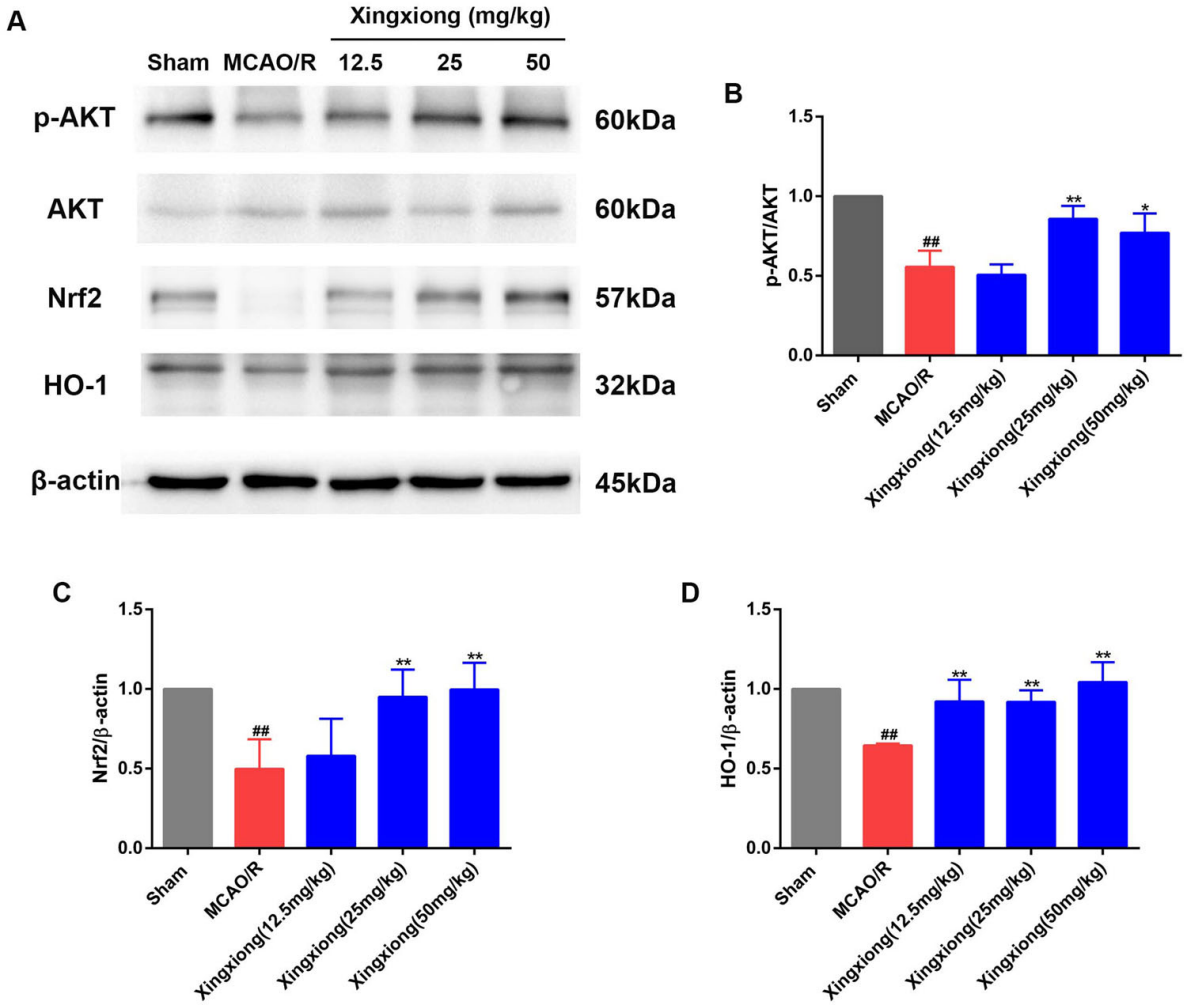
Xingxiong injection reduces oxidative stress by activating the Akt/Nrf2/HO-1 pathway. Representative images of immunoblotting (A) and quantification of the relative protein levels of p-AKT/AKT (B), Nrf2 (C) and HO-1 (D) in the infarct cortex region of the different groups on day 14 after MCAO surgery. *n* = 3 in each group. Data are expressed as the mean ± SD and were analysed by ANOVA. ^##^*p* < 0.01 vs. sham group; *p < 0.05, **p < 0.01 vs. MCAO/R group.

In view of the fact that HO-1 is an important antioxidant enzyme, we examined cortex HO-1 protein expression. Figure 4 shows that MCAO/R rats had lower HO-1 expression level than rats in the sham group. However, Xingxiong injection treatment for 14 d significantly increased HO-1 expression level. Nrf2 directly regulates the activity of HO-1 promoter. Western blot results reveal that Nrf2 expression level was higher in rats treated with Xingxiong injection than rats in the MCAO/R group. Moreover, activation of the PI3K/Akt signal transduction pathway could promote Nrf2 nuclear translocation (Zhai et al. 2018). We aimed to assess if PI3K/Akt signal transduction pathway is involved in the mechanisms underlying the Xingxiong injection-mediated reduction of oxidative stress. Western blot results also reveal that the expression level of phospho-Akt (p-Akt) were higher in rats pre-treatment with Xingxiong injection than rats in the MCAO/R group, indicating that Xingxiong injection could promote Akt phosphorylation in the cortex region.

### Suppression of ischaemia/hypoxia-induced inflammation by Xingxiong injection *in vivo* and *in vitro*

**Figure 5.**
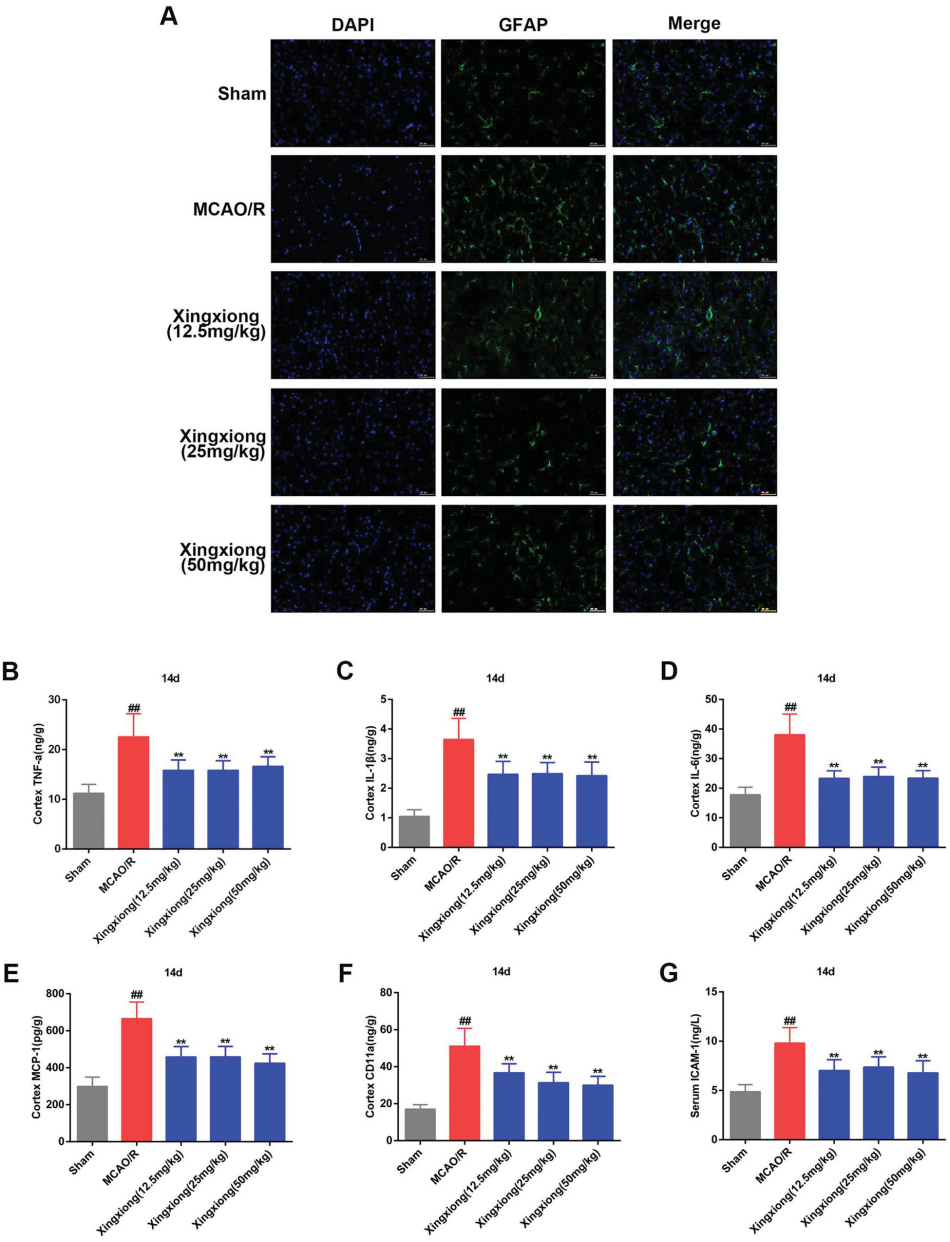
Xingxiong injection inhibits inflammatory response in rat cortex with MCAO/R. (A) Immunofluorescence for GFAP (14 days postreperfusion) in the cortex. Scale bar: 50 μm. Effect of Xingxiong injection treatment on cortex TNF-α (B), IL-1β (C), IL-6 (D), MCP-1 (E), CD11a (F) and ICAM-1 (G) in rats with MCAO/R. Data are expressed as the mean ± SD and were analysed by ANOVA. ^##^*p* < 0.01 vs. sham group; **p < 0.01 vs. MCAO/R group.

Cerebral ischaemia promotes the activation and proliferation of astrocytes, and the reactive astrocytes produce and release inflammatory mediators, such as IL-6, TNF-α and IL-1β (Liu and Chopp 2016; Pekny et al. 2016). In the present study, the increased reactive astrocytes that were labelled with GFAP was observed in the MCAO/R-induced cortex region were attenuated by Xingxiong injection (Figure 5(A,B)).

**Figure 6.**
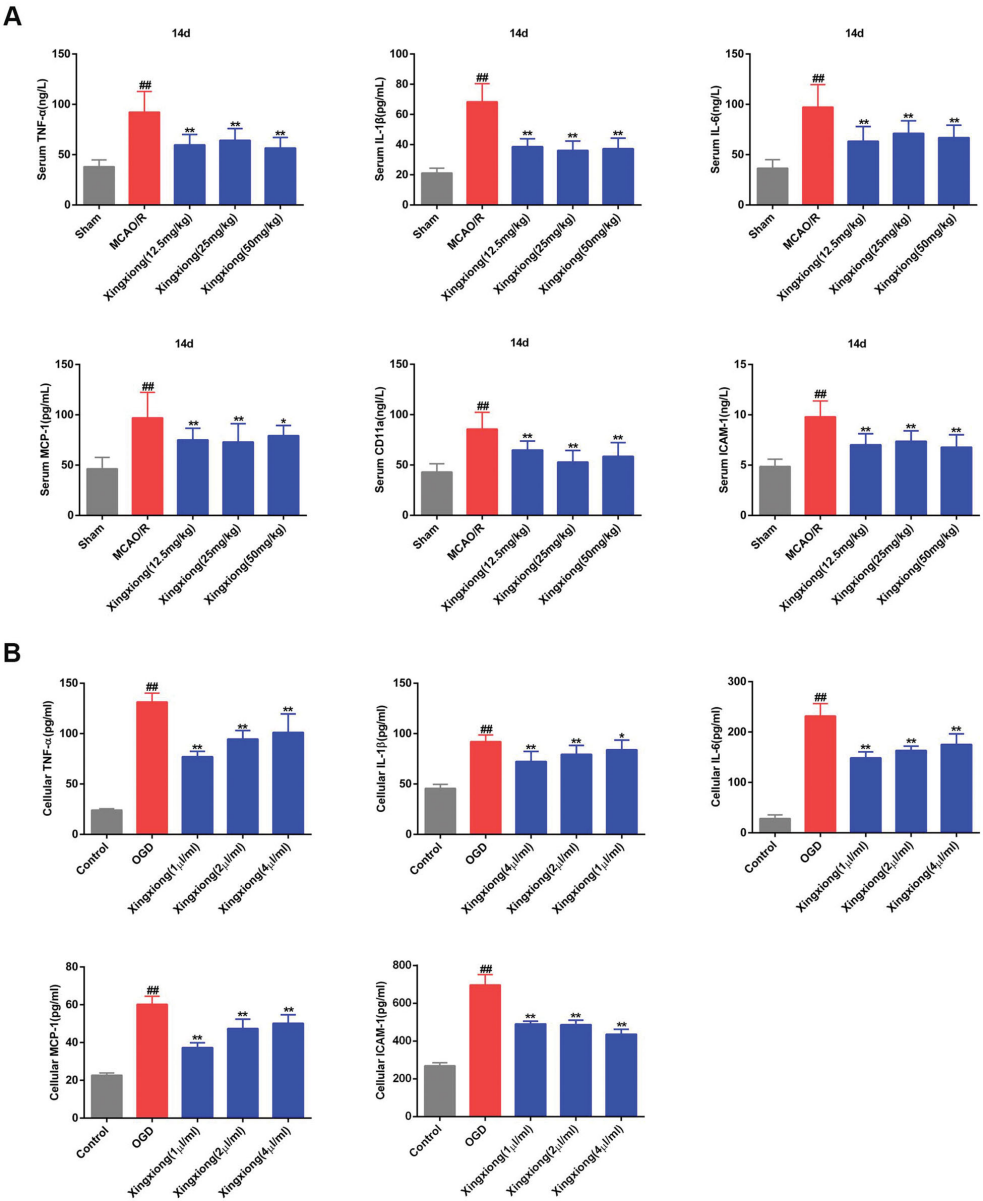
Xingxiong injection inhibits the expression of inflammatory factors in rat serum with MCAO/R and in primary cortical neurons with OGD/R. Effect of Xingxiong injection treatment on TNF-α, IL-1β, IL-6, MCP-1, CD11a and ICAM-1 in rat serum (A) with MCAO/R (*n* = 8) and in primary cortical neurons (C) with OGD/R (*n* = 3). Data are expressed as the mean ± SD and were analysed by ANOVA. ^##^*p* < 0.01 vs. sham or control group; *p < 0.05, **p < 0.01 vs. MCAO/R and OGD/R group.

Results of ELISA of ischaemia/hypoxia-induced inflammation *in vivo* and *in vitro* are shown in Figures 5(C) and 6. The expression of excessive inflammatory factors, such as TNF-a, IL-1β, IL-6, MCP-1, CD11a and ICAM-1 was induced by ischaemia in rat cortex (Figure 5(B)) and serum (Figure 6(A)) after MCAO surgery for 14 d. There excessive inflammatory factors with the exception of CD11a were up-regulated expressed in the OGD-treated primary cortical neurons *in vitro* (Figure 6(B)). However, the expression levels of these inflammatory cytokines in the cortex and serum of MCAO-treated rats and in OGD-treated primary cortical neurons were inhibited by Xingxiong injection.

### Xingxiong injection inhibits NLRP3 inflammasome activation

**Figure 7.**
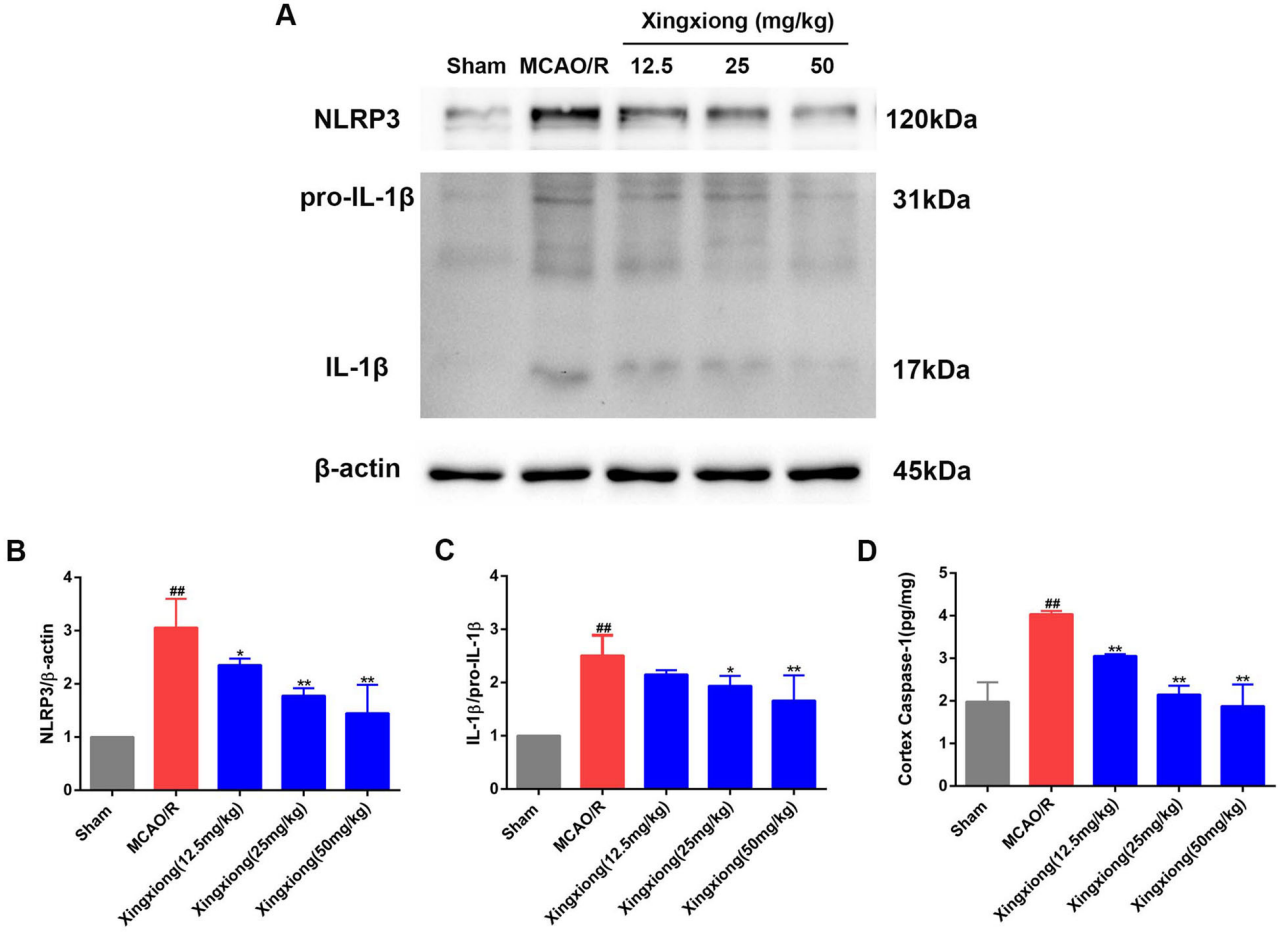
Xingxiong injection reduces inflammatory response by inhibiting NLRP3 inflammasome. Representative images of immunoblotting (A) and quantification of the relative protein levels of NLRP3 (B) and IL-1β (C) in the infarct cortex region of the different groups on day 14 after MCAO surgery, *n* = 3 in each group. Effect of Xingxiong injection treatment on caspase-1 in cortex region (D) with MCAO/R by ELISA (*n* = 3). Data are expressed as the mean ± SD and were analysed by ANOVA. ^##^*p* < 0.01 vs. sham group; *p < 0.05, **p < 0.01 vs. MCAO/R group.

As shown in Figure 7(A–C), the active IL-1β protein expression level was observably upregulated in the cortex of MCAO/R rats, which suggested activation of NLRP3 inflammasome in the cortex. In addition, result of ELISA of ischaemia-induced caspase-1 expression level in cortex tissue is shown in Figure 7(D). The result shows that the caspase-1 expression level in cortex was significantly decreased in the Xingxiong injection-treated rats compared with the MCAO/R-treated rats. Therefore, we detected the protein expression level of NLRP3 in the cortex. As expected, NLRP3 expression level in the cortex was significantly increased in MCAO/R-treated rats compared with the rats in the sham group. However, rats pre-treated with Xingxiong injection exhibit a significant decrease in NLRP3 expression level in the cortex compared with the MCAO/R group.

## Discussion

Ischaemic stroke was mainly caused by cerebral ischaemia and reperfusion injury (Jamison et al. 2008; Xie et al. 2018). After ischaemic stroke, the pathophysiological responses of the brain were predominated by BBB perturbations, free radical production, brain oedema and excitatory amino acid toxicity in the early acute phase of ischaemic injury, followed by inflammation and neuronal apoptosis in the middle stage, and then angiogenesis and neurogenesis, which provide the critical neurovascular substrates for neuronal remodelling, in the delayed phase (Arai et al. 2009). In the present study, MCAO/R rat model was used to explore the effects and mechanisms of Xingxiong injection following acute ischaemic stroke. Results show that infarct volume, neurological deficit, neuronal injury, neuronal apoptosis and glial activation were improved in the MCAO/R-treated rats by Xingxiong injection treatment. Moreover, the Xingxiong injection-treated rats exhibited decreased NOX, protein carbonyl, 4-HNE, 8-OHdG and various inflammatory factors compared with the MCAO/R group. In addition, we also found that Nrf2 pathway and NLRP3 inflammasome were involved in the neuroprotective effects of Xingxiong injection against ischaemic stroke *in vivo*. These results indicated that activation of Nrf2 pathway and inhibition of NLRP3 inflammasome may be the molecular mechanisms against oxidative stress and inflammation after ischaemic stroke challenge.

Mitochondria, one of the most important organs in brain neurons, played an important role in regulating Ca^2+^ homeostasis in cytoplasm, maintaining intracellular pH and generating ATP through the tricarboxylic acid cycle and electron transport chains. Disorders of ROS and Ca^2+^ overload can cause the integrity of the mitochondrial inner membrane to be damaged, the mitochondrial membrane potential (Δψm) depolarized and decreased, and the proteinic pores (mitochondrial permeability transition pore, MPTP) in the membrane to open. Subsequently, numerous small molecules (relative molecular mass <1.5 kDa) pass through the membrane to increase the osmotic pressure of mitochondrial matrix proteins, resulting in mitochondrial outer membrane damage and mitochondrial membrane potential depolarization. These changes further induce mitochondrial apoptosis and activate caspase cascade (Jonas et al. 2015; Mnatsakanyan et al. 2017). The activation of caspase-3 pathway caused by ischaemia would induce neurons apoptosis, thereby leading to the neurological dysfunction. The present study demonstrated that Xingxiong injection treatment reduced TUNEL positive cells, increased mitochondrial membrane potential and lowered the activity of caspase-3. Therefore, inhibiting apoptosis might be an important mechanism of Xingxiong injection treatment for playing a role of neuroprotective effect.

Cerebral I/R injury induced the expression of NOX, PC, 4-HNE and 8-OHdG, which might represent the response of oxidative stress suffering from ischaemic insult. Oxidative stress refers to a condition in which the imbalance favouring ROS/RNS over antioxidative ability causes cellular injury and pathologic change (Zhou et al. 2018). ROS/RNS are mainly generated in the ischaemic penumbra, particularly after reperfusion and are continuously removed through powerful free radical scavenging systems (e.g., SOD, GSH, CAT, GSH-Px and NADP/NADPH) to maintain a dynamic balance (Zhou et al. 2019). Under the condition of ischaemia/reperfusion, increased generation of active free radicals can induce lipid peroxidation, protein denaturation, intracellular Ca^2+^ overload, enzyme inactivation and DNA damage and mitochondrial damage, which promotes cytochrome-C (Cyt-C) and apoptosis inducing factor (AIF) in mitochondria release, cellular signalling pathway disorder and downstream apoptotic cascade reaction (Sherif and Al-Shaalan 2018). In addition, oxidative stress exacerbates the inflammatory response (Bahar et al. 2017) and triggers the activation of matrix metalloproteinases (Ito et al. 2017), thereby disrupting the integrity of BBB and accelerating neuronal apoptosis and white matter damage. The present study has shown that Xingxiong injection treatment significantly inhibits the increased levels of oxidative stress markers (such as NOX, protein carbonyl, 4-HNE and 8-OHdG) induced by MCAO/R in rats *in vivo* and OGD/R in primary cortical neurons *in vitro*. These results indicated that the neuroprotective effects of Xingxiong injection were partly caused by oxidative stress inhibition.

HO-1 is a powerful antioxidant that catalyses the degradation of biliverdin, iron and carbon monoxide. Inducing the expression of HO-1 can enhance the antioxidant stress ability of neurons (Meng et al. 2014b). Previous studies have shown that transcription factor Nrf2 is a key factor in regulating the expression of HO-1, which is regulated by PI3K/Akt (Martin et al. 2004).

In our study, higher expression levels of p-Akt/Akt, Nrf2 and HO-1 were observed in rats treated with Xingxiong injection than in the MCAO/R group, indicating that Xingxiong injection treatment activates the Akt/Nrf2 pathway and promotes HO-1 expression.

Inflammatory cascades, triggered by energy depletion and necrotic neuron death play an important role in ischaemia/reperfusion process. Inflammatory response is activated by blood-borne leukocytes that penetrate the brain during the ischaemic process (Kamel and Iadecola 2012). Ischaemia/reperfusion injury induces ROS production, glutamate toxicity, Ca^2+^ overload, platelets and endothelial cell activation, and releases inflammatory cytokines and pro-inflammatory mediators including TNF-a, IL-1β, IL-6, ICAM-1, CD11a, MCP-1 and anti-inflammatory mediators including IL-4, IL-10 in ischaemic brain (Boehncke 2018). TNF-α is a potent stimulator of microglial activation closely related to inflammatory response. TNF-α aggravates the inflammatory response after cerebral ischaemia, promotes vascular endothelial cells damage and thrombosis formation. TNF-α is also an important promoter of inflammatory factors, which can upregulate the expression of other inflammatory factors (such as IL-1β, IL-6, IL-8, etc.) and intercellular adhesion factor-1 (ICAM-1), promoting the transfer of inflammatory cells to nervous tissue and aggravating neurological dysfunction (Gong et al. 2019). In our research, MCAO/R injury significantly deteriorated neurological function. These phenomena were accompanied by the enhanced expression of a series of pro-inflammatory cytokines such as TNF-a, IL-1β, IL-6, ICAM-1, CD11a and MCP-1. In contrast, Xingxiong injection inhibits all above-mentioned activities induced by MCAO/R challenge both *in vivo* and *in vitro* study. The increased protein level of ICAM-1 observed from the serum and cortex following MCAO/R challenge is consistent with previous research manifesting that high ICAM-1 level was more likely to have previous stroke and increased haemorrhagic transformation risk (Wu et al. 2018). This increase was triggered by MCAO/R challenge and inflammatory cytokine overproduction, which was dramatically limited by Xingxiong injection treatments, proved that Xingxiong injection reduced cerebral ischaemia/reperfusion injury by inhibiting inflammatory response.

Inflammasomes play an important role in the inflammatory response after ischaemic stroke. Among them, NLRP3 is the most representative inflammasomes, which has become a research topic of great interest. After ischaemic stroke, DAMPs released by necrotic cells in the ischaemic core and changes in intracellular microenvironment can trigger the formation of inflammasomes complexes, activating caspase-1 and mediating the secretion of IL-1β and IL-18, and finally cause cell death through inflammation and/or apoptosis. Western blot analysis confirmed that Xingxiong injection downregulates the protein expression of NLRP3 and IL-1β/pro-IL-1β. ELISA results also show that Xingxiong injection significantly downregulates the caspase-1 expression levels in cortex and serum. The data suggest that Xingxiong injection exerts anti-inflammatory effects after ischaemic stroke via the inhibition of NLRP3 activation.

## Conclusions

The current study showed that treatment with Xingxiong injection inhibits apoptosis, oxidative stress and inflammation after ischaemic stroke. The mechanisms by which Xingxiong injection exert antioxidant and anti-inflammatory effects involved the upregulation of Akt/Nrf2 pathway and inhibition of NLRP3 inflammasome. These findings clarified the mechanism underlying the function of Xingxiong injection for the clinical treatment of ischaemic stroke.
